# Microglia-induced neuroinflammation in hippocampal neurogenesis following traumatic brain injury

**DOI:** 10.1016/j.heliyon.2024.e35869

**Published:** 2024-08-08

**Authors:** Seyedeh Parisa Navabi, Firuzeh Badreh, Maryam Khombi Shooshtari, Somayeh Hajipour, Sadegh Moradi Vastegani, Seyed Esmaeil Khoshnam

**Affiliations:** a Persian Gulf Physiology Research Center, Medical Basic Sciences Research Institute, Ahvaz Jundishapur University of Medical Sciences, Ahvaz, Iran; b Behbahan Faculty of Medical Sciences, Behbahan, Iran

**Keywords:** Traumatic brain injury, Neuroinflammation, Microglia, Neurogenesis, Hippocampus

## Abstract

Traumatic brain injury (TBI) is one of the most causes of death and disability among people, leading to a wide range of neurological deficits. The important process of neurogenesis in the hippocampus, which includes the production, maturation and integration of new neurons, is affected by TBI due to microglia activation and the inflammatory response. During brain development, microglia are involved in forming or removing synapses, regulating the number of neurons, and repairing damage. However, in response to injury, activated microglia release a variety of pro-inflammatory cytokines, chemokines and other neurotoxic mediators that exacerbate post-TBI injury.

These microglia-related changes can negatively affect hippocampal neurogenesis and disrupt learning and memory processes. To date, the intracellular signaling pathways that trigger microglia activation following TBI, as well as the effects of microglia on hippocampal neurogenesis, are poorly understood. In this review article, we discuss the effects of microglia-induced neuroinflammation on hippocampal neurogenesis following TBI, as well as the intracellular signaling pathways of microglia activation.

## Highlights

• Mitochondrial dysfunction and mtROS following TBI triggers inflammation cascades in microglia through inflammasome.
• Microglia play key roles in activating and regulating inflammatory signal transduction pathways in different TBI models.
• Neuro-inflammatory pathways in microglia hinder neuronal stem cell differentiation and proliferation in the hippocampus.
• Several compounds can improve microglia inflammation and hippocampal neurogenesis by modulating neuro-inflammatory pathways.

## 1 Introduction

Traumatic brain injury (TBI) as one of the most common causes of death in the world, leads to neurological dysfunction through primary and secondary mechanisms [1]. The focal or diffuse patterns of primary effects characterized by neuronal and vascular damage, myelin loss and reactive gliosis which intensifies by secondary effects, such as mitochondrial dysfunction, disruption of calcium homeostasis, edema, reductions in ATP levels, and apoptosis pathways depending on severity of injuries [2,3]. The primary and secondary injury processes eventually cause neuronal damage, which can be the beginning of disorder in different areas of the nervous system [4].

Studies have shown that TBI alters hippocampal neurogenesis, causing acute loss of newborn neurons and excessive proliferation of progenitor cells [5]. Neurogenesis takes place within a distinct region of the adult hippocampus known as the subgranular zone (SGZ), situated between the hilus of the dentate gyrus (DG) and the granular layer, housing mature neural progenitor cells [6]. Following the division of these progenitor cells, newborn neurons are generated, subsequently migrating to the DG and undergoing differentiation into adult granule cells [7]. The precise extent of adult neurogenesis in the human hippocampus remains uncertain; however, it is estimated to be approximately 30 % of the hippocampal neural network in rodents [8]. The hippocampus is very vulnerable to TBI due to its structure and function. Although not all models of TBI directly cause damage to the hippocampus, most studies have reported the presence of signs of injury following TBI [9]. Consequently, TBI has been shown to adversely impact adult neurogenesis in the hippocampus, thereby contributing to the onset of neurodegenerative conditions such as memory and learning disorders [10].

Many post-traumatic neurological disorders are mediated through inflammatory responses [11]. Inflammatory mediators, which are produced by activated microglia and reactive astrocytes, have been identified as major contributing factor for neurodegenerative conditions [12]. Microglia are macrophage-like cells derived from yolk sac erythro-myeloid precursors that migrate to and distribute throughout the central nervous system (CNS) [13]. Upon exposure to injurious stimuli, microglia initiate an inflammatory response by releasing inflammatory mediators [14]. Additionally, Pro-inflammatory factors have been shown to suppress mitochondrial function. In this context, several studies have underscored the importance of the mitochondria in the processes of survival and differentiation of neural progenitor cell (NPC) in the SGZ [15,16] of the hippocampal DG and SVG [17]. However, innate proliferation of NPCs occur after TBI, but it is insufficient for compensation of cell loss, especially under conditions of inflammation and oxidative stress [18]. Consequently, several therapeutic approaches have progressively focused on post-injury neurogenesis to improve TBI outcomes [19]. In this review, we examine microglia-induced neuroinflammation on hippocampal neurogenesis following TBI. Additionally, we discuss the role of intracellular signaling pathways and mitochondrial dysfunction in microglia activation and polarization.

## 2 TBI pathogenesis

TBI is a physical injury to brain tissue resulting from an external mechanical force, leading to temporary or permanent neurological deficits [20]. The neurological damages associated with TBI are categorized into two groups: Primary damages caused by mechanical forces at the same time as the insult, and secondary damages that occur in the long term after the trauma [21]. Studies have demonstrated that in cases of moderate to severe TBI, both primary and secondary injuries manifest [22]. Depending on the severity of the injury, patients may experience various symptoms, including cognitive, behavioral, mood, and motor deficits, which can impact their abilities and quality of life [23].

Following TBI, a cascade of cellular and molecular mechanisms is initiated that exacerbate its effects, including oxidative stress, blood-brain barrier (BBB) disruption, neuroinflammation, mitochondrial dysfunction, axonal degeneration, and neuronal apoptosis [24]. BBB disruption resulting from TBI triggers the migration of activated leukocytes into the damaged brain parenchyma [25]. Activated leukocytes and microglia contribute to cytoskeleton disruption and axonal demyelination by producing ROS and inflammatory factors such as cytokines and chemokines [26]. As a result, synaptic transmissions are disturbed, and this axonal damage eventually leads to neurodegeneration [21]. Mitochondrial dysfunction is also implicated in caspase-dependent neuronal apoptosis through the release of cytochrome *c* and apoptosis-inducing factors [27,28].

## 3 Adult hippocampal neurogenesis

Numerous studies have demonstrated that neurogenesis occurs in both the hippocampal DG and subventricular zone (SVZ) of lateral ventricles following neurodegenerative diseases such as ischemia, targeted cortical cell ablation, seizure, and various TBI models, including controlled cortical impact (CCI), diffuse traumatic axonal injury, fluid percussion injury (FPI), and closed head injury (CHI) [29]. Additionally, the migration of newly generated cells to the injured brain region has been observed in these conditions [30].Various stages of neurogenesis are estimated by measuring the following markers in the adult hippocampus: Nestin (expressed in precursor cells), Ki-67 (expressed in neuroblasts), DCX (expressed in immature neurons), BrdU (expressed in newborn neurons), and NeuN (expressed in mature neurons) [31]. However, the percentage of differentiation and maturation to new neurons remains typically low due to excessive neurotoxic molecules, inflammation, and lack of trophic signals [32].

Previous studies have shown that moderate-severe CCI enhances BrdU incorporation into newborn neurons as early as 24 h post-injury in several brain regions, including the injured cortex, SVZ, and DG of the hippocampus, which continues for seven days [33]. However, most of the newly generated neurons do not survive, and only some of these newborn neurons express NeuN [5].

Different TBI models show contradictory effects on neurogenesis depending on the severity of injuries. For example, Wang et al. showed that a mild CCI model did not affect neurogenesis, while moderate TBI only stimulated neural stem cells (NSCs) proliferation, and severe TBI increased neurogenesis at all three stages (NSC proliferation, immature neurons, and mature neurons) [10]. NSCs possessing high differentiation potential into new neurons, oligodendrocytes, and astrocytes, play an important role in neural regeneration in neurodegenerative conditions mainly located in two regions of the adult brain: the SGZ [17] and the SVZ [34].

It has been suggested that in moderate TBI, more than 50 % of resident immature neurons are reduced in the SGZ, which is compensated for by proliferation and differentiation of NSCs in the hippocampus [35,36]. These new neurons play a significant role in cognitive tasks [37,38]. Similar to animal models, the expression of NSC protein markers such as DCX, PSA-NCAM, TUC4, SOX2, and NeuroD was also increased in the perilesional cortex of the human brain after TBI compared to the normal brain [38]. Therefore, inducible activation of NSCs can be used as a novel approach to ameliorate TBI-induced disorders [39].

## 4 Role of microglia in adult hippocampal neurogenesis

Neurogenesis has garnered increasing attention in neurotrauma experiments due to its potential therapeutic role in neural regeneration after injuries. However, the mechanisms regulating neurogenesis after pathological brain injuries remain to be elucidated, and the prominent role of microglia in neurogenesis should not be ignored. In this regard, the potential role of microglia in adult neurogenesis in TBI models also remains unknown [40].

Microglia, as resident immune cells in the brain, morphologically divide into ramified microglia in the healthy CNS and reactive microglia in pathologic condition [41]. Briefly, microglia could regulate cell morphology, cell migration, phagocytosis, and inflammatory response through the expression or production of some mediators such as: 1) Pattern recognition receptors (PRRs) (toll-like receptors (TLRs), nucleotide-binding oligomerization domain (NOD)-like receptors (NLRs), leucine-rich repeat (LRR)-containing receptors, and retinoic acid-inducible gene-1 (RIG-1)-like receptors (RLRs)), 2) Cytokine receptors and cytokine production (tumor necrosis factor-alpha (TNF-α), transforming growth factor-beta (TGF-β), and interleukins), 3) Chemokine receptors (CCR) and chemokine production (C chemokines, CC chemokines, and CX3C chemokines). Among these ligands/receptors, the CX3CL1 (CX3C ligand 1, exclusively expressed in neurons)/CX3CR1 (exclusively expressed in microglia) pathway particularly plays an important role in neuron-microglia communication [42,43].

In the intact brain, ramified microglia regulate adult hippocampal neurogenesis through several mechanisms: 1) increasing the proliferation, migration and differentiation of NPCs in the SGZ, 2) producing growth factors such as insulin-like growth factor 1 (IGF-1) and brain-derived neurotrophic factor (BDNF), and 3) eliminating most NPCs dying in the first few days of their life by phagocytosis [44,45]. Conditioned media containing NPCs and microglia have been shown to have a higher proportion of neuronal differentiation and survival compared to cell culture without microglia [46]. In addition, microglia interact with nearby healthy neurons through the CX3CL1 (Fractalkine)/CX3CR1 signaling pathway to maintain a ramified phenotype and regulate hippocampal neurogenesis. It has been suggested that the reduction in fractalkine signaling may result from increased neuroinflammation, leading to decreased hippocampal neurogenesis [47].

Interestingly, it has been shown that the direct infusion of BDNF, a key factor in neuroblast migration, as well as growth factors into the adult rat hippocampus, significantly enhances BrdU-labeled neurons in the granule layer following a TBI model [5,48]. In line with this, by changing the microglia phenotype toward microglial-expressed BDNF, researchers could achieve prevention of CCI-induced reduction in hippocampal neurogenesis [49]. Thus, microglia in healthy and pathological situations differentially affect hippocampal neurogenesis. For example, healthy microglia promote neurogenesis through phagocytosis of dysfunctional and apoptotic progenitors, support of progenitor migration, synaptic maintenance, secretion of trophic and anti-inflammatory factors, as well as regulation of the CX3CL1/CX3CR1 pathway [42,50]. While pathologically activated microglia prevent neurogenesis through impaired phagocytosis of apoptotic and dysfunctional progenitors, failure of synaptic maintenance, secretion of inflammatory and cytotoxic factors, and dysregulated CX3CL1/CX3CR1 pathway, previous experiments revealed that induction of microglia activation (for example, lipopolysaccharide injection) predominantly inhibits the survival of new cells [42,51]. The comparison between ramified microglia and activated microglia regarding neurogenesis is illustrated in Fig. 2. Conversely, reduction of microglia activation (minocycline or indomethacin treatment) stimulates hippocampal neurogenesis after some neurodegenerative diseases such as epilepsy, cranial irradiation, and ischemic stroke. In contrast with these reports, a recent TBI model by Ng et al. showed that indomethacin, with a 50 % successful decrease in reactive microglia, didn't support neurogenesis in the hippocampus, lateral ventricles, or pericontusional cortex in all stages, possibly due to insufficient reduction of activated microglia [32]. Another TBI model revealed that upregulation of miR-124 contributes to augmenting ramified microglia, increasing BrdU/NeuN in the DG region of the hippocampus, which positively affects neurogenesis [52].

## 5 Microglia activation and neuroinflammation

Microglia can be categorized into classical (M1, with an amoeboid shape, round, and large cell bodies, neurotoxic) or alternative (M2, with a small cell body, distal branches, neuroprotective) phenotypes [53]. Microglia, depending on the surrounding environment, can switch to another polarization type and may have either inflammatory or anti-inflammatory effects on the CNS, described as a double-edged sword. However, the pattern of microglia activation varies, and there are several intermediate phenotypes (satellite, Keratan sulfate proteoglycan (KSPG), Hoxb8, CD11c, and dark microglia) between them based on proliferation style, chemotaxis, phagocytosis, migration, and cytokine secretion [54]. Therefore, the M1/M2 paradigm is a simplified theory and an inadequate idiom to designate microglia activation accurately. In this way, it has been said that under physiological conditions, the quiescent phenotype (M0) shows an “immune defense” role, which can switch to both M2 phenotype in mild injury (through “find me” signals from the injury site) or M1 phenotype in severe injury (through “eat me” signals from the injury site) [55].

Overall, M1 microglia produce inflammatory factors such as interleukins (IL-6, IL‐1β, or IL‐12), interferon-γ, tumor necrosis factor-alpha (TNF-α), adenine dinucleotide phosphate oxidase (resulting in the production of superoxide and reactive oxygen species), proteolytic enzymes (metalloproteinases, inducible nitric oxide synthase, heme oxygenase 1), CC chemokine ligands, costimulatory molecules (CD36, CD45, CD47), and integrins (CD11b, CD11c), which contribute to neurological damage [56,57]. Meanwhile, M2 microglia produce anti-inflammatory cytokines (transforming growth factor, IL-10), fibroblast growth factor, growth factors, neurotrophic growth factors (BDNF), and pro-survival factors, promoting cell debris phagocytosis to support tissue repair and neuron survival [58]. Many modulators are reported to be involved in this microglia polarization, including different types of receptors, signal transduction pathways, transcription factors, cytokines, chemokines, ion channels, drugs, bioactive compounds, and so on [59]. In this regard, maintaining the balance between microglia M1/M2 phenotypes or shifting microglia towards M2 polarization may present interesting ideas as potential therapeutic approaches in neurodegenerative diseases [60,61].

### 5.1 Intracellular signaling pathways triggers microglia activation

**Fig. 1:**
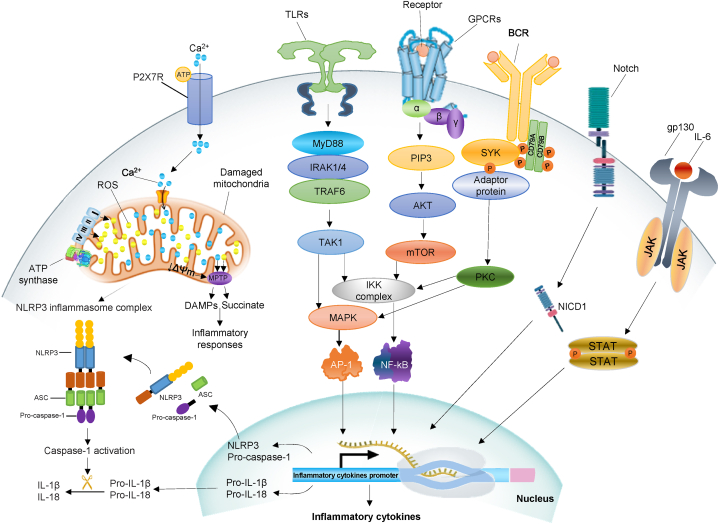
Schematic overview of various intracellular signaling pathways associated with activation of microglia and production of inflammatory cytokines. In one of these signaling pathways, toll-like receptors (TLRs) activate myeloid differentiation factor 88 (MyD88), which induces production of inflammatory cytokines through downstream pathways of mitogen-activated protein kinases (MAPKs), activator protein-1 (AP-1), and nuclear factor kappa-light-chain-enhancer of activated B cells (NF-κB).In the P2X7 receptor (P2X7R)-dependent signaling pathway, influx of Ca2+ ions increases mitochondrial Ca2+ level, overproduction of adenosine triphosphate (ATP) and reactive oxygen species (ROS), and induces mitochondrial membrane potential (ΔΨmt) collapse, which consequently activates NLRP3 inflammasome and Inflammatory responses. G protein-coupled receptors (GPCRs) act as regulators of microglial activation that activate NF-κB through the phosphatidylinositol 3-kinases/protein kinase B (PI3K/AKT) signaling pathway and phosphorylation of IκB Kinase Complex (IKK). Splenic tyrosine kinase (SYK) is one of the intracellular regulator of microglial activation that is activated by the B cell receptor (BCR), leading to phosphorylation and activation of the Protein kinase C (PKC) signaling pathway. Notch signaling is involved in microglia activation following notch ligand binding and release of notch intracellular domain (NICD).

Signaling pathways governing the TBI-induced brain inflammation have not been fully elucidated. Activation of microglial receptors is mostly accomplished through a variety of intracellular pathways, such as Janus kinase-signal transducer and activator of transcription suppressors of cytokines (JAK/STAT/SOCS), phosphatidylinositol 3-kinase (PI3K)/AKT/mTOR), Akt/GSK-3β/CREB, toll-like receptors4/nuclear factor kappa B (TLR4/NF-kB), CaMKKβ-dependent AMPK/PGC-1α, RAF/MEK/ERK, purinergic 2X7 receptor (P2X7R) and spleen tyrosine kinase (SYK) pathways [62,63]. The intracellular signaling pathways involved in the production of inflammatory cytokines and the activation of microglia are shown in Fig. 1. Additionally, the compounds involved in the regulation of multiple signal transduction pathways following TBI are listed in Table 1.

**Fig. 2:**
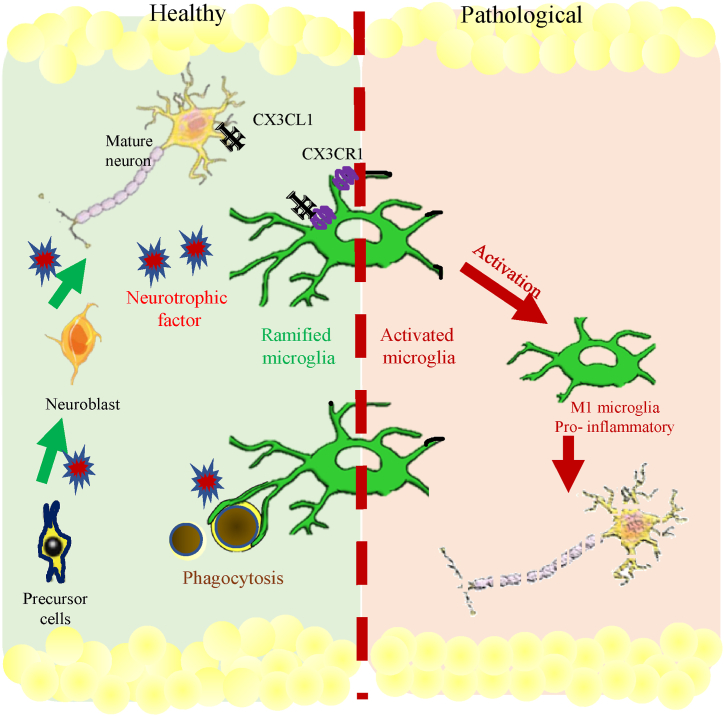
Schematic representation comparing ramified microglia and activated microglia in neurogenesis. In the healthy brain (left side), microglia promote neurogenesis through phagocytosis of dysfunctional and apoptotic progenitors (brown circle), support of progenitor migration, synaptic maintenance, secretion of trophic factors (red star), and communication with nearby neurons through the CX3CR1/CX3CL1 signaling. Interactions between ligands and substrates contribute to the ability of microglia to maintain a surveillant/ramified phenotype. In pathological conditions (right side), activated microglia hinder neurogenesis through impaired phagocytosis of apoptotic and dysfunctional progenitors, synaptic maintenance failure, and secretion of pro-inflammatory and cytotoxic factors. This signaling results in a change in microglia phenotype and function, leading to decreased neurogenesis.

**Table 1:** Compounds involved in regulation of multiple signal transduction pathways following TBI.

Molecules	TBI models	Pathways	Properties	Ref.
Mer	CCI model	STAT1/SOCS Signaling	Important regulator of microglial/macrophage M1/M2 polarization and neuroinflammation	[64]
AG490	FeeneyTBI model	JAK2/STAT3 pathway	Involved in the neurological function recovery after TBI.	[65]
RNF6	CCI model	JAK/STAT3 pathway	Alleviated TBI by Inhibition of RNF6 and suppressing the STAT3 signaling	[66]
Sevoflurane	FeeneyTBI model	PI3K/AKT pathway	Reduce neuronal apoptosis by autophagy inhibiting and Attenuate TBI	[67]
Dex	weight-drop device	PI3K-AKT-TOR pathway	Suppress autophagy	[68]
Tetrahydrocurcumin	CCI model	PI3K/Akt pathway	Reduce apoptosis through modulation autophagy	[69]
Simvastatin	CCI model	PI3K/Akt Pathway	Restoration of cognitive function after TBI	[70]
FTY720	weight-drop device	PI3K/Akt pathway	Exerte neuroprotective effects and autophagy	[71]
Salidroside	CCI model	PI3K/Akt pathway	Reduce apoptosis	[72]
17Beta-estradiol	traumatic cerebral contusion	Akt pathway	inhibit the activation of caspase-3, activates ERK1/2 and Akt at the early stage	[73]
ω-3 PUFA	FeeneyTBI model	HMGB1/TLR4/NF-κB pathway	Inhibit TBI-induced microglial activation	[74]
Progesterone	CCI model	TLR4/NF-κB pathway	Express proinflammatory cytokines	[75]
Resveratrol	CCI model	TLR4/NF-κB pathway	reduces neuronal autophagy and inflammatory reactions	[76]
Apocynin	CCI model	TLR4/NF-κB pathway	module neuronal autophagy	[77]
hADSC-ex	weight-drop device	NF-κB pathway	Inhibit TBI-induced microglia/macrophage activation	[78]
mGlu5 PAM		Akt/GSK-3β/CREB	reduce pro-inflammatory microglial activation in vitro	([79])
CSF1	transgenic mouse model	CREB signaling	provide powerful neuroprotective and survival signals in brain injury	([80])
Glycyrrhizin (GL)	rat models of TBI	HMGB1/TLR4/NF-κB	inhibit the expression and release of HMGB1 after TBI	([77])
Curcumin	FeeneyTBI model	TLR4/MyD88/NF-κB	decrease in microglia/macrophages, inflammatory factor release	([81])
Balasubramide		CaMKKβ-dependent AMPK/PGC-1α	suppress neuroinflammation in vitro and in vivo by modulating microglial activation state	([82])
Trametinib	CCI model	MEK/ERK	suppresses microglia-induced neuroinflammation after TBI animal	([83])

Abbreviations: AKT, Akt kinase; CCI model, controlled cortical impact model; CSFI, Colony-stimulating factor 1; Dex, Dexmedetomidine; HMGB1, High-mobility group box 1; JAK, Janus kinases; Mer, Myeloid-epithelial-reproductive tyrosine kinase; NF-κb, nuclear factor (NF)-κB; ω-3 PUFA, Omega-3 polyunsaturated fatty acid; PI3K, Phosphoinositide 3-kinase; STAT, signal transducers and activator of transcription; SOCS, suppressors of cytokine signaling; TLR4, Toll-like receptor 4.

#### 5.1.1 JAK/STAT/SOCS signaling pathway

JAK/STAT signaling pathway is closely associated with brain injury and has been shown to be activated after TBI. This pathway plays a significant role in anti-inflammatory responses and several other biological processes [84]. There are four JAK tyrosine kinases: JAK1, JAK2, JAK3 and Tyk2 (Non-receptor tyrosine-protein kinase TYK2); and seven STAT transcription factors: STAT1, STAT2, STAT3, STAT4, STAT5a, STAT5b and STAT6 in mammals [85]. Suppressors of cytokine signaling (SOCS) proteins act as a negative regulator of JAK/STAT signaling and are key physiological regulators of inflammation [86,87].

Recently, the important role of SOCS in macrophage function has been reported. SOCS1 has been reported to play a crucial role in regulating macrophage polarization and function [88]. Changes in macrophages have been shown to be influenced by epigenetic mechanisms in which SOCS1 acts as a capacitor for M1/M2 polarization [89]. Upregulation of DNA methyltransferase 1 (DNMT1) appears to be associated with hypermethylation of SOCS1, activation of the JAK2/STAT3 signaling pathway in macrophages and LPS-induced proinflammatory cytokines (TNF-α, leading to the release of IL-6) [90]. These results suggest that loss of SOCS1 expression may induce inflammatory M1 action in macrophages via activation of the JAK/STAT signaling pathway [89].

Both the function and mechanism of SOCS-2 have been reported to involve the SHP2 binding site of the activated growth hormone (GH) receptor, attenuating GH signaling by inhibiting JAK2 and STAT5b axis activation [91]. SOCS2 is believed to be a major controller of macrophage activation. Genetic studies using a transgenic approach have shown that SOCS2-overexpressing transgenic (SOCS2Tg) mice had increased numbers of CD11b + CD206 + M2 macrophages compared to wild-type littermates after mild or moderate TBI, suggesting functional improvement in anti-inflammatory responses [92]. As expected, M1 population was enriched in SOCS2(−/−) mice, and the altered polarization coincided with enhanced IFN-γ-induced STAT1 activation in SOCS2(−/−) macrophages [93]. Additionally, SOCS3 protein is well known to act as a negative regulator of STAT3 (a key physiological regulator in immune homeostasis and disease pathogenesis) [94].In fact, SOCS3 is a cytokine signaling repressor that may inhibit the expression of inflammatory genes in macrophages [95]. There is several evidences suggesting that SOCS3 is involved in modulating macrophages M1/M2 polarization by the transcriptional and post-transcriptional mechanisms [96].

#### 5.1.2 PI3K/Akt/mTOR signaling pathway

The PI3K-AKT/mTOR signaling pathway is composed of two main parts: Phosphatidylinositol-3-kinase (PI3K) and its downstream serine/threonine protein kinase, known as AKT, and mammalian/mechanistic target of rapamycin (mTOR) [97,98]. PI3Ks can be categorized into three specific classes: class I, class II and class III. PI3Ks of class I, are directly activated by tyrosine phosphorylation of cell surface receptors. There are three key subtypes of the main molecule downstream of the PI3K signaling pathway, AKT1, AKT2 and AKT3 [99,100]. AKT1 is highly expressed in many tissues, AKT2 is mainly expressed in insulin-sensitive tissues and is present at low levels in other tissues, and AKT3 is only expressed in the brain and testis [101]. The specific tissue expression patterns of the various AKT subtypes suggest that they play important roles in maintaining physiological function in different tissues [102,103].

mTOR is a member of the PI3K-related kinase protein family that is expressed in different brain regions (16–18). mTOR includes two distinct functional complexes: mTOR complex 1 (mTORC1) and mTOR complex 2 (mTORC2) [104]. Previous evidence has shown that mTOR is a direct substrate for the Akt kinase and has identified Serine 2448 as the Akt target site on mTOR [105]. The PI3K/Akt/mTOR signaling pathway is involved in the expression and production of pro-inflammatory mediators [106,107] and microglial activation [108] in neurological conditions such as brain trauma. The fact that PI3K/Akt/mTOR signaling pathway plays a crucial role in multiple biological activities including cell growth, proliferation and cell survival [109]. Experimental evidence shows that inhibition of this signaling pathway with lipopolysaccharide (LPS) leads to a reduction in the level of proinflammatory factors by microglia activation [107]. Conversely, Activation of PI3K/Akt has been reported to participate in the protection of brain damage by the inhibition of inflammation and apoptosis [110].

#### 5.1.3 Akt/GSK-3β/CREB signaling pathway

In this pathway, Akt phosphorylates the constitutively active serine-threonine kinase GSK-3β at Ser-9 [111], resulting in GSK-3β inhibition [112]. GSK-3β activation is associated with neurodegeneration, and is known to affect transcription factors involved in inflammatory activation [113,114]. Among these transcription factors, CREB - a basic leucine zipper (bZIP) transcription factor - is activated by Akt/GSK-3β signaling [115] to regulate key inflammatory genes, such as IL-10 and TNF-α [116], through GSK-3β inhibition [115].

#### 5.1.4 TLR4/NF-kB signaling pathway

The other signaling pathway that related to neuronal damage after TBI is Toll-like receptor 4 (TLR4)/nuclear factor (NF)-κB signaling pathway [74]. Toll–like receptor 4 (TLR4), a member of the pattern –recognition receptor family, acts as a pathogen/damage molecular patterns in several cell type such as microglia, astrocyte and neurons in the CNS, inducing an inflammatory downstream cascade in response to brain injuries such as trauma, Alzheimer's, or stroke [117]. This receptor activates downstream signaling pathways, such as the nuclear factor (NF)-κB signaling pathway and its cascades, leading to the production of inflammatory cytokines and chemokines [118,119], via the gene expression of mediators such as interleukin-1β (IL- 1β), tumor necrosis factor-α (TNF-α) [120], interleukin-6 (IL-6), intercellular adhesion molecule-1 (ICAM-1), and monocyte chemoattractant protein-1 (MCP-1) [121]. Then, the activation of TLR4 induces NF-κB-dependent apoptosis, expression of proinflammatory factors [122,123], and autophagic neuronal death [121]. However the exact molecular mechanisms underlying autophagy need to be explored further.

#### 5.1.5 CaMKKβ-dependent AMPK/PGC-1α signaling pathway

The other signaling pathway is the CaMKKβ-dependent AMPK/PGC-1α pathway. The AMP-activated protein kinase (AMPK), a conserved serine/threonine kinase, serves as a cellular energy sensor maintaining homeostasis and regulating metabolic pathways [124], including metabolic and functional changes of neurons in brain damage [125]. AMPK can be phosphorylated and activated in response to increase in the intracellular AMP-to-ATP ratio [126], and it has been shown to be associated with the transition of microglia/macrophages from M1 to M2 [127,128]. As a result, it markedly reduces inflammation and inhibits tissue inflammatory damage in various models [129]. Calcium/calmodulin-dependent protein kinase kinase-β (CaMKKβ) is the upstream activator of AMPK, whose activation regulates microglia-induced neuroinflammation [130].

#### 5.1.6 RAF/MEK/ERK signaling pathway

Another pathway that has been implicated in microglial pro-inflammatory responses is the Mitogen-activated protein kinase (MEK)/extracellular signal-regulated protein kinase (ERK) pathway. In fact, the MEK/ERK pathway is important and required for MAPK signal transduction [131,132]. The MAPK signaling transduction pathway is also known as the Ras-Raf-ERK MAPK pathway. In mammals, the MAPK/ERK pathway includes seven transduction families [79]. All families share two serine/threonine kinases and one bispecific threonine/tyrosine [133]. ERK, a member of the MAPK family, which also comprises the MEK1 and MEK2 genes, plays a key role in signaling cascades and transmits extracellular signals to intracellular sites [80]. This pathway is often activated in certain diseases, and the activation of the MAPK/ERK pathway promotes cell aging, apoptosis, and inflammatory responses [134]. Recently, the MEK inhibitor, trametinib has been shown to efficiently suppress microglia-induced neuroinflammation after TBI animal, providing a potentially effective strategy for TBI patients [135].

The MAPK signaling pathway is activated in innate immune cells such as dendritic cells and macrophages as a defense mechanism by TLRs and various cytokines [136]. The MAPK signaling cascade consists of three protein kinases with sequential action: At the beginning of the pathway, there is a MAP3K [also known as MEKK or MKKK] that is phosphorylated and activates the downstream factor MAP2K [also known as MEK or MKK], and finally, the MAPK factor [also known as ERK] is phosphorylated and activated at the end of this pathway [137]. This pathway leads to the expression of the matrix metalloproteinase (MMP) gene, resistance to apoptosis, and cell survival through the activation of many transcription factors such as NF-kB [138].

#### 5.1.7 Notch signaling pathway

The Notch signaling pathway, which is involved in cell-cell contact procedures, encompasses four Notch receptors (NOTCH 1–4) and five ligands of the Delta-Serrate-Lag (DSL) family. The interaction between a transmembrane Notch receptor and a transmembrane ligand of a neighboring cell leads to proteolytic cleavage and the separation of the Notch intracellular domain (NICD), which translocates to the nucleus and acts as a transcriptional co-activator to promote the expression of target genes. This process is involved in stem cell maintenance, proliferation, differentiation, and microglia polarization [60].

#### 5.1.8 P2X7R signaling pathway

P2X receptors (P2XRs) are ATP-gated nonselective cation channels composed of seven subtypes (P2X1–7) and have equal permeability to Na+, K+, and Ca2+ under physiological conditions [139]. Activation of the receptor through the binding of three ATP molecules leads to the rapid opening of the channel and the passage of small ions across the cell membrane [140]. In the central nervous system, P2X7 receptor expression is increased under neuroinflammatory situations in microglia, astrocytes, and oligodendrocytes and also is involved in cerebral neurological damages and edema following TBI or in conditions that cause an increase in extracellular ATP content [141].

#### 5.1.9 SYK signaling pathway

Spleen tyrosine kinase (SYK) is a non-receptor protein tyrosine kinase involved in modulating immune signals in various cells including mast cells, B cells, and macrophages [142]. Activation of Syk leads to the activation of downstream molecules, including MAPK, PI3K, AKT, and NF-κB, leading to the production of proinflammatory and inflammatory mediators by B-cells, mast cells, and macrophages [143,144].

#### 5.1.10 Cell death following microglia activation

Cellular autophagy is the process of destruction and recycling of cellular components by the cell itself. Autophagy can be triggered by traumatic brain injury; however, the role of autophagy in TBI is controversial. Some studies suggest the protective role of autophagy after TBI [145,146], while numerous studies indicate the detrimental role of autophagy after TBI [[77], [81], [147], [148], [149]]. In the latter case, activation of some signaling pathways reduces apoptosis by regulating autophagy in TBI. As mentioned above, the activation of the PI3K/AKT/mTOR pathway provides neuroprotection in TBI via inhibition of autophagy.

Regarding the relation between members, mTORC1 (which consists of mTOR, Raptor, GLβ and some other proteins) acts as a negative modulator of autophagy [82], and the PI3K/AKT signaling pathway acts as a major regulator of mTORC1 [150]. AKT activates mTOR via direct phosphorylation of tuberous sclerosis complex 2 (TSC2) at the serine residue located at position 939 [151]. Phosphorylation of TSC2 leads to Rheb activation, which promotes activation of the mTOR kinase activity [83,152]. Hence, AKT inhibitors suppress mTOR activity, leading to dephosphorylation of Beclin-1 regulator 1 (AMBRA1), activation of unc-51 like autophagy activating kinase 1/2 (ULK1/2) complex, phosphorylation of focal adhesion kinase family interacting protein 200 (FIP200), and subsequent autophagy initiation [153].

Research has demonstrated that Nrf2, a redox-sensitive transcription factor containing a basic leucine zipper domain, possesses the ability to modulate the process of autophagy [154,155]. Zhang et al. [156] found that autophagy after TBI is regulated by Nrf2 and provide neuroprotection, although this connection it not well understood. There are several possible explanations based on the role of p62. P62 binds to ubiquitin chains and leads to the initiation of autophagy [157]. In addition, another study showed that Keap1 uncoupled from Nrf2 can bind to p62, interact with LC3, and transport the conjugated ubiquitin to autophagosome for the degradation process [158].

The mechanism by which Nrf2 regulates autophagy requires further investigation. Secondary injury after TBI can be reduced by FoxO3a, which belongs to the forkhead transcription factor family [159]. This factor has also been confirmed to regulate autophagy. To date the effects of Foxo3a on autophagy have only been presented in TBI models by directly increasing the transcription of autophagy-related proteins (ATGs), such as Beclin-1, LC3, Atg5 and Atg7 [160].

The other factor that regulates autophagy in brain injury models such as TBI is TLR4. A recent study showed that genetic ablation of TLR4 could actively inhibit autophagy and thereby mitigate the neuroinfammatory response after TBI [147]. Also, a NF-kB binding site has been reveal in the Beclin-1 promoter. Therefore, NF-kB activation by TLR4 can up-regulate the expression of Beclin-1 and promote autophagy [161]. This evidence suggests that inhibition of autophagy acts as a potent agent to attenuate TBI-induced brain inflammation.

### 5.2 Mitochondrial dysfunction triggers microglia activation

Mitochondria play a pivotal role in cell survival, growth, and death. They not only contribute to cellular energy provision via ATP synthesis but also play an essential role in buffering intracellular calcium, producing mitochondrial ROS (mtROS), and regulating apoptosis [[162], [163], [164], [165], [166], [167]]. Mitochondria participate in nearly all cell functions, and their dysfunction is particularly involved in the pathogenic mechanisms following TBI [1]. A growing body of literature has shown that mitochondrial dysfunction is an early event and one of the hallmark events after TBI, contributing to metabolic and physiologic dysregulations that lead to cell death [[168], [169], [170]]. In the experimental models of TBI, mitochondrial dysfunction occurs within 24 h post-injury and can last up to 14 days, resulting in reduced ATP production in cortical and hippocampal tissues [[170], [171], [172]].

Several mechanisms have been suggested for TBI-induced mitochondrial dysfunction. Briefly, intracellular calcium accumulation and the influx of excessive ions into mitochondria result in mtROS production, mitochondrial membrane depolarization, and inhibition of ATP synthesis [21,170,173]. Subsequently, the mitochondrial electron transport chain (ETC) is disrupted, and oxidative phosphorylation processes are impaired, leading to the breakdown of metabolic reactions required for cell survival and intracellular calcium balance [[174], [175], [176]]. These conditions also lead to the opening of the mPTP (mitochondrial permeability transition pore), which increases the permeability of the mitochondrial inner membrane [177,178].

Mitochondrial proteins such as cytochrome *c* (cytc) and AIF (apoptosis inducing factor) are released to the cytosol and play key role in apoptotic cell death [170,179]. In addition, mitochondrial dysfunction activates the NLRP3 (Nod-like receptor protein 3) inflammasome, which increases the production of pro-inflammatory cytokines such as IL-1β and IL-18 [17,180]. These studies highlight the prominent role of mitochondria in TBI pathophysiology. Therefore, ameliorating mitochondrial function may induce neuroprotection and improve cognition following TBI [168,169]. There is increasing evidence that mitochondrial dysfunction and inflammation are related and exacerbate each other during neurological disorders such as Alzheimer's disease [[181], [182], [183]] Parkinson [184], ischemic stroke [[185], [186], [187]], multiple sclerosis [188], amyotrophic lateral sclerosis [189], and TBI [190].

#### 5.2.1 NLRP3 inflammasome following mitochondrial dysfunction

It has been increasingly suggested that the NLRP3 inflammasome serves as a conduit between mitochondrial dysfunction and pro-inflammatory signaling in the microglial cells [191,192]. Inflammasomes can be activated in response to various infectious or sterile stimuli, such as pathogenic microbes (e.g., parasites, fungi, bacteria, and viruses) and host-derived danger signals (e.g., mitochondrial dysfunction, ROS, ion flux, and metabolic factors), through germline-encoded pattern recognition receptors (PRRs) present on the membrane or within the internal compartments of innate immune system cells [193,194].

Activation of inflammasome sensors (NAIP/NLRC4, NLRP3/6/7, AIM2/IHI16) is usually followed by the initiation of the canonical inflammasome complex through recruitment and formation of pro-caspase-1 filaments [195,196]. Non-canonical inflammasomes are assemblies that activate caspase-11/-8 in mice and caspase −4/-5 in humans. As a result, active caspase-1 or caspase-8 mediate the processing of the inflammatory cytokines IL-1β and IL-18 into mature form. Active caspase-1/-11-/4/-5 can induce pyroptosis or activate the NLRP3 inflammasome through GSDMD cleavage [197].

The NF-κB transcription factor acts as a pivotal mediator of inflammation and innate immunity and is composed of an inactive complex consisting of three subunits: IκB, P65, and P50 [198]. A main regulator of NF-κB signaling pathways is the IκB kinase (IKK) complex, which phosphorylates IκB, leading to the translocation of P50 and P65 to the nucleus, resulting in increased transcription of anti-apoptotic and pro-inflammatory genes [199].

NLRP3 inflammasome activation is a two-step process involving priming and activation. In the first, or priming, step, activation of the NF-κB pathway through TLR and P2X7R receptors leads to up-regulation of NLRP-3, pro-IL-1β and pro-IL-18 proteins. In the second or activating step, oligomerization of NLRP3 occurs through the NACHT domain, which after the polymerization of ASC causes the recruitment and activation of pro-caspase-1. Activated caspase-1 induces cleavage/processing of the cytokines IL-1β and IL-18 for inflammatory responses [200,201].

Mitochondrial dysfunction plays a contributory role in the activation of the NLRP3 inflammasome in the microglial cells [202]. There is evidence that microglia-derived NLRP3 inflammasome is involved in the inflammatory response to TBI, as the use of MCC950 (a selective NLRP3 inflammasome inhibitor) reduces the post-TBI inflammatory response and BBB disruption [203]. While activation of the NLRP3 inflammasome is beneficial for the innate immune responses to the pathogens and tissue injury [204], its overactivity leads to a form of cell necrosis known as pyroptosis [205]. Evidence shows that inhibition of the NLRP3 inflammasome can alleviate neuroinflammation and improve outcomes following TBI. In addition, circulating NLRP3 and its related molecules could be used as potential biomarker for neuroinflammation after TBI [206].

It has recently been shown that extracellular mitochondria (exMTs) released from injured cerebral cells can promote inflammation following TBI [207]. The exMTs is a major source of DAMPs (damage-associated molecular patterns), which could activate the innate immune system and initiate inflammatory responses [[208], [209], [210], [211]]. Several mechanisms contribute in exMTs-induced inflammation. The exMTs can promote the activation of neutrophils and increased its interaction with endothelium [212]. The exMTs released by activated monocytes could induce pro-inflammatory factors such as type I interferon and TNF-α responses in endothelial cells, thus promote inflammation [213]. ExMTs, produced by platelets, serve as a substrate for bactericidal group IIA-secreted phospholipase A_2_ to promote inflammation [214].

It has also been reported that release of mtDNA (oxidized mitochondrial DNA), mtROS, and cardiolipin can activate the NLRP3 inflammasome [[215], [216], [217], [218]], which discussed below. Activation of the NLRP3 inflammasome stimulates caspase-1 [219,220], which leads to activation and release of IL-1β and IL-18 [221], thus inducing further inflammation [222]. These mitochondrial-related danger signals are initially translated into adaptive responses, and if homeostatic processes cannot be re-established, cell death is executed [223].

Releasing mtDNA from injured mitochondria activates NLRP3 inflammasomes [224,225], and mtROS has been suggested to play a pivotal role in mtDNA release [226]. Additionally, during apoptosis, mtDNA is oxidized by mtROS and released into the cytosol, where it binds to and activates the NLRP3 inflammasome, resulting in increased inflammation [227]. In fact, cytosolic oxidized mtDNA is associated with the NLRP3 inflammasome complex and is required for its activation [228]. The NLRP3 inflammasome induces mitophagy, a process by which cells can clear damaged mitochondria [229]. However, the process of mitophagy in macrophages attenuates NLRP3 inflammasome activity by clearing mtDNA from the cytosol [226].

MtROS was demonstrated to increase after mitochondrial dysfunction, promoting the activation of the NLRP3 inflammasome [230], and could be a direct activator of the NLRP3 inflammasome [216]. In addition to its activator role, mitochondria have also been reported as a docking system for inflammasome assembly [231]. This role of mitochondria is due to the externalization of cardiolipin to the outer membrane, which can interact with pro-caspase-1 and NLRP3 [232]. It has also been suggested that cardiolipin directly binds to the LRRs (leucine-rich repeats) of NLRP3, activating this inflammasome [218].

#### 5.2.2 Cell death following mitochondrial dysfunction

Neuronal and glial cell death, along with traumatic cell injury, participate in the pathogenic mechanisms during TBI [168]. Overall, mitochondrial dysfunction initially activates several devastating cascades after TBI, eventually leading to extensive neuroinflammation and cell death. Three major forms of cell death—apoptosis, necrosis, and autophagy [233]—contribute to cell damage following TBI [168]. Mitochondria are associated with various modes of cell death, and their membrane permeability releases death factors [[234], [235], [236]].

One of the hallmarks of TBI-induced secondary brain injury is apoptotic cell death of neurons and oligodendrocytes [237,238], which can be triggered by the activation and interaction of different pathways, including p38, MAPK, ERK (extracellular signal-regulated kinase), and JAK/STAT (janus kinase/signal transducer and activator of transcription) [239,65]. Mitochondrial dysfunction can induce apoptotic cell death through caspase-dependent mechanisms, known as the intrinsic pathway of apoptosis after TBI [240]. This pathway is activated when cytC is released after mPTP opening, which increases inner membrane permeability and releases pro-apoptotic molecules into the cytoplasm [187,241]. Opening of the mPTP, caused by calcium accumulation, mtROS, and mitochondrial depolarization [[242], [243], [244], [245], [246]], leads to the release of cytC and the initiation of apoptotic cascades [[247], [248], [249], [250]]. CytC forms an ATP-dependent complex with apaf-1(apoptotic-protease-activating factor-1) in the cytosol, triggering caspase-dependent downstream signaling through activation of caspase 8 and 9, ultimately leading to caspase 3 activation [[251], [252], [253], [254]]. Besides, caspase-independent apoptosis in TBI can be activated by the release of mitochondrial proteins such as AIF, HtrA2/OMI (high-temperature requirement protein A), endonuclease-G, SMAC/DIABLO (second mitochondrion-derived activator of caspase/direct inhibitor of apoptosis-binding protein with low pI), alteration of the mitochondrial ETC and cellular redox homeostasis, and loss of mitochondrial transmembrane potential, ultimately causing DNA damage [[255], [256], [257], [258]]. Apoptotic cell death is profoundly regulated by anti-apoptotic proteins such as the B-cell lymphoma-2 (Bcl-2) family and death-inducing factors such as Bcl-2-associated X protein (BAX) [259]. Several studies show that the expression of Bax and BCL-2 proteins is upregulated after TBI, triggering mitochondria-associated apoptotic pathways in traumatic brain [260,261].

Induction of mPTP opening, in addition to the apoptosis process, is involved in inducing autophagic and necroptotic cell death, and increasing protein levels of necroptotic markers (RIPk3 and RIPk1) and autophagy markers (LC3-II/LC3-I ratio and Beclin-1) [262]. It is well-established that the pro-inflammatory M1 phenotype of microglia is associated with enhanced TBI pathogenesis, whereas a switch toward the M2 phenotype is associated with recovery processes [263].

During neuroinflammation, the M1 to M2 switch in microglia may hold beneficial therapeutic potential, as mitochondrial function may determine this microglia differentiation [264]. Activated microglia and brain-infiltrating immune cells can alter mitochondrial metabolism, mtROS formation, and programmed cell death by producing inflammatory mediators such as TNF [264]. Indeed, activated microglia inhibits OXPHOS (oxidative phosphorylation) through TNF production, inducing enhanced mitochondrial ROS [265]. Pro-inflammatory cytokines shift ATP production from OXPHOS to glycolysis in mitochondria, providing the energy required for changing cell function and survival [264]. For example, succinate from the inactive Krebs-cycle can trigger IL-1β production via HIF1-α (hypoxia-inducible factor 1-alpha) [266]. Overall, the activation of the Inflammasome NLRP-3 in pathologic conditions is mediated by two pathways: mitochondrial dysfunction and the activation of multiple intracellular signaling pathways in the M1 phenotype of microglia [267,268].

## 6 Potential therapeutic compounds toward TBI recovery: the role of microglia

Myeloid-epithelial-reproductive tyrosine kinase (Mer), a member of the Tyro-Axl-Mer (TAM) family of receptor tyrosine kinases [269], plays a key role in the regulation of macrophage inflammatory responses [270] and brain microglial cells [271]. In a recent study, researchers demonstrated that induction of STAT1/SOCS signaling in the brain tissues of TBI-injured mice via Mer activation leads to increased M2-like polarization of microglia/macrophages [64].

Moreover, Raible et al. suggested that the severity of injury affects the magnitude of JAK/STAT pathway activation in animal model of TBI [272]. In this study, by inhibiting the activation of the JAK2/STAT3 pathway in TBI rats, TBI symptoms were ameliorated after administration of AG490. Another study showed that RNF6 inhibition can suppress the activation of STAT3 by JAK [66]. The neuroprotective effects of Dexmedetomidine (Dex) have been studied in rats exposed to TBI. When Dex was administered to TBI rats, concomitantly with increased levels of *p*-Akt and *p*-mTOR, decreased levels of microtubule-associated protein 1 light chain 3 (LC3) and Beclin-1 were observed. These results suggest that Dex can suppress autophagy via the activation of the PI3K-AKT-TOR pathway [68]. In addition, several studies on neuroprotective factors such as tetrahydrocurcumin [69], simvastatin [70], FTY720 [71], salidroside [72], and estradiol [73] have reported that activation of the PI3K/AKT pathway has potential therapeutic effects for TBI.

However, emerging evidence has suggested that PI3K/AKT pathway activation plays a critical role in anti-apoptotic and anti-autophagy responses for neuroprotection after TBI [273]. A recent study found that inflammation induced by TBI in rodents was decreased by mGlu5 PAM treatment, whose signaling mechanism involved the Akt/GSK-3β/CREB pathway [274]. Although CREB can be activated by different signaling pathways, including ERK1/2, cAMP/PKA, and PI3K/Akt [275]. Reports from experimental and clinical studies have proven that Omega-3 polyunsaturated fatty acid [74], progesterone [75], resveratrol [76], apocynin [77], Ciprofloxacin and levofloxacin [276] play neuroprotective roles in TBI and improve outcomes following TBI by attenuating the TLR4/NF-κB signaling pathway [277].

Research has also shown that high mobility group box one (HMGB1) activates neuroinflammation through receptors such as Toll-like receptor (TLR2) and TLR4, as well as the receptor for advanced glycation end products (RAGE). This study shows that glycyrrhizin (GL) attenuates inflammatory responses, at least partly, by inhibiting HMGB1/TLR4/NF-κB signaling pathway in a rat model of TBI [278]. HMGB1 can bind directly to the TLR4/MD-2 complex to induce the release of proinflammatory cytokines in macrophages [279]. Recently, a study showed that stem cell-derived exosomes enter microglia/macrophages and suppress their activation during brain injury [78]. For example, human adipose mesenchymal stem cell-derived exosomes suppress the neuroinflammatory activation of macrophages and reduce neuronal apoptosis by inhibiting NFκB and P38 mitogen-activated protein kinase signaling in an LPS-induced inflammatory model [78]. Furthermore, in the TLR4 signaling pathway, Zhu et al. showed that curcumin treatment after LPS stimulation significantly decreased the release of inflammatory mediators such as IL-1β, IL-6, and RANTES, and suppressed the protein expressions of microglial TLR4/MyD88/NF-κB signaling pathway [280].

There are several therapeutic approaches that increase the proliferation, differentiation and survival of NSCs in the brain. For example transplantation of NPCs could improve neuronal differentiation and survival, synaptic plasticity, hippocampal neurogenesis, spatial memory, and immune modulation after TBI [281], which may be mediated through PI3K/AKT signaling pathway [282]. The hippocampus, as the most vulnerable area to brain injury, exhibits atrophy, deficits in long-term potentiation (LTP), protein expression, and alterations in hippocampal signaling which are correlated with memory deficit in TBI models and other neurodegenerative diseases [[283], [284], [285], [286]]. Interestingly, induction of LTP at the perforant pathway to the DG [17] could promote NPCs proliferation and the rate of neurogenesis due to strong depolarization and glutamate activation of many signaling pathways [287]. However, the main limitation of the therapeutic approach of stem cells in TBI is the poor viability and low differentiation of transplanted stem cells [288].

Bone marrow-derived mesenchymal stem cells/sodium alginate/collagen type I/stromal cell derived factor-1 (BMSCs/SA/Col/SDF-1) scaffold promotes the migration of BMSCs to the lesions and partly increases neurogenesis by activating the SDF-1/CXCR4-mediated FAK/PI3K/AKT and probably GSK3β/β-catenin pathway [288]. Microglia can also promote hippocampal proliferation and survival through the secretion of neurotrophic factors, communication with neighboring neurons, and removal of apoptotic newborn cells by ramified microglia [289].

In comparison with microglia, astrocytes exhibit a long-lasting proliferative response, lasting at least 28 days after TBI. Astrocytes produce the mitogenic protein S100β in vivo, which induces improvement in hippocampal neurogenesis, learning, and memory in rat models of TBI [290,291]. Astrocytes remarkably express factors such as neurotrophic factors and gliotransmitters, signal transducer and activator of transcription-3 (STAT3), and TLR, which support neuronal integrity and mediates anti-inflammatory responses [292]. Astrocytes also play a crucial role in neurogenesis, synaptogenesis, angiogenesis, BBB integrity, synaptic plasticity, anti-inflammatory reactions, axonal regeneration, and synaptic remodeling after TBI [291]. However, the molecular and cellular mechanisms related to the correlation between neurogenesis and TBI are not fully understood [3].

N -acetyl serotonin (NAS), as a tropomyosin related kinase receptor B (TrkB) agonist, exerts both antioxidant and anti-apoptotic activity through the TrkB/PI3K/Akt/CREB signaling pathway in NPCs. It increases hippocampal neurogenesis, expression of phosphorylated CREB in the DG region, and cognitive performance in a mouse model of CCI [19]. Activation of the Akt/GSK3beta/beta-catenin signaling pathway delays neuronal apoptosis, a major cause of post-traumatic mortality, which may be mediated by mitochondrial cytochrome c-induced caspase 3 release [293]. Some therapeutic compounds involved in mitochondrial regulation following TBI are also specified in Table 2.

**Table 2:** Therapeutic compounds involved in the regulation of mitochondria following TBI.

Compound	TBI models	Pathways	Properties	Ref.
BAY 11-7082	CCI	NLRP3 inhibitor	Improved memory performance and brain tissue histology, reduced edema, BAX, IL-1b and Caspase-1 and increased Bcl-2 and BCL-XL protein expression	[294]
Cyclosporin A	TBI	mPTP inhibitor	Improving mitochondrial membrane potential. Modulation of calcium homeostasis and oxidative damage. Improve cognition and strengthen neuroprotection	[295]
GDF11	ICH	phosphorylation of mitochondrial ETC	Modulation of mitochondrial fission and fusion. Attenuating mitochondrial dynamic abnormality and dysfunction	[296]
Olaparib	CCI	PARP inhibitor	Improving mitochondrial function and preventing neuronal cell death. Increasing mt-DNA repair	[297]
SS-31	TBI	SIRT1 and PGC-1α	Reversal of mitochondrial dysfunction. Reduction of ROS content, malondialdehyde level and release of cytochrome *c*. Preventing the reduction of superoxide dismutase activity.	[298]
MitoQ	mild closed TBI	Nrf2	Increased activity of antioxidant enzymes Reducing the content of malondialdehyde (MDA) Decreased transfer of Bax protein to mitochondria and release of cytochrome *c* into the cytosol	[299]
N-Acetylcysteine Amide	spinal cord injuries	–	Decreasing oxidative stress Increasing mitochondrial bioenergetics normalizing GSH levels	[300]
AdipoRon	TBI	Adiponectin/AdiopR1, SIRT3, AMPK-PGC	Mediating mitochondrial homeostasis and antioxidant system	[301]
Mitoquinone	TBI	Nrf2/ARE HO-1 and NQO-1	Increased expression of cytosolic Bax protein compared to the mitochondria Increased expression of mitochondrial cyt *c* compared to cytosol	[302]
Resveratrol	TBI	ROS/GSK-3β	Decreased GSK-3β activation and mitochondrial damage Reduce glutamate-induced cytotoxicity	[303]
Polydatin	TBI	p-PERK, XBP-1, ATF6, SIRT1, p38	Reducing the release of reactive oxygen species in neuronal mitochondria Reduction of mitochondrial swelling Maintain mitochondrial membrane potential Opening of mitochondrial permeability transition pore	[304]

Abbreviations: WT, wild type; CCI model, controlled cortical impact model; NLRP3, nucleotide binding oligomerization domain-like receptor family pyrin domain containing 3 or inflammasome.

In a study, the beneficial effects of electroacupuncture (EA) on hippocampal neurogenesis were demonstrated by the increase of double-labeled newborn neurons located in the SGZ of the DG in TBI model. These effects are probably exerted through the inhibition of both the TLR4/Myd88/NF-ĸB and TLR4/TRIF/NF-ĸB signaling pathways post-trauma [305]. However, it has been shown that TLR4 is expressed on the surface of NSCs, and stimulation of the TLR4 pathway suppresses the ameliorative effects of EA on hippocampal neurogenesis, neurocognitive, and neurobehavioral recovery after TBI [34,39].

As mentioned, post-TBI neuroinflammation negatively affects hippocampal NSCs and delays recovery. Moreover, the M1 microglia phenotype impairs hippocampal neurogenesis, while the M2 microglia phenotype improves hippocampal neurogenesis. In this regard, treatment with exosome-miR-124 after TBI promoted M2 polarization of microglia (inhibiting the TLR4 pathway), increased hippocampal neurogenesis (enhancing NSCs proliferation and differentiation), as well as improved neural function and cognition in behavioral tests [306]. Exosomes are small membrane-enclosed vesicles derived from certain pluripotent cell types such as MSCs [307], potentially ameliorating hippocampal neurogenesis and neuroinflammation relative to MSCs in TBI models [308].

The critical immune-modulatory receptor family, sphingosine 1- phosphate receptors (S1PRs), is highly expressed in hippocampal NSCs and SVZ, which might be responsible for biological changes in NSCs such as differentiation and migration toward the injured area. It is reported that S1PRs are essential for hippocampal NSCs differentiation through MEK/Erk phosphorylation after post-traumatic conditions [39]. A variety of proteins, such as vascular endothelial growth factor (VEGF), BDNF, CREB, and Na + -K + -2Cl− co-transporter type 1 (NKCC1), have been demonstrated to be involved in post-TBI neurogenesis [309]. Promotion of hippocampal neurogenesis and survival of neonatal neurons by overexpression of suppressor of cytokine signaling 2 (SOCS2) may be mediated through BDNF and its receptor TrkB, which is associated with the increased anti-inflammatory phenotype of M2 macrophages after TBI [2].

Two pathways are involved in the neurogenic effect of VEGF in the hippocampus, as a hypoxia-induced neurotrophic and angiogenic factor after TBI: the PI3K–Akt pathway that promotes cell survival through anti-apoptic, proangiogenic, and neuroprotective activity, and the Raf/MEK/ERK pathway through DNA synthesis and cell growth. VEGF, especially through VEGF receptor-2 and VEGFR-3 receptors, mediates axonal elongation, cell proliferation and chemotaxis in glial precursor cells [3,310]. NKCC1, which is highly expressed in neurons and astrocytes has been documented that regulate neuronal volume, ion homeostasis, cell proliferation, and neuroblast migration. In this way, upregulation of NKCC1 is correlated with the activation of Raf/MEK/ERK cascade, upregulation of HIF-1α and VEGF, and adult hippocampal neurogenesis [309].

The Wnt/β-catenin signaling pathway, through the proliferation and differentiation of NPCs in both SVZ and SGZ, is important for the recovery of neural injuries such as TBI model. Surviving, the downstream target gene of the Wnt/β-catenin pathway, induces neurogenesis in immature and mature neurons in the DG region of the hippocampus in a time-dependent manner after the induction of TBI [[311], [312], [313]]. The Raf/MEK/ERK and Wnt/β-catenin pathways have been reported to be involved in differentiation and proliferation [314]. Traumatic spinal cord injury (SCI) or neurotrauma is associated with varying degrees of axonal demyelination, permanent sensorimotor loss, and altered conductivity of ion channels [315]. In this regard, regeneration process after SCI requires activation of the Wnt/β-catenin signaling pathway to act directly on radial glia and induce their differentiation into adult neurons [316,317].

The Notch signaling pathway is a developmental process that participates in various key processes such as survival, differentiation, proliferation, function, and homeostasis [318]. Moreover, Notch signaling is involved in the M1/M2 imbalance in RA [319]. Notch signaling involves in neurogenesis through the induction of quiescent NSCs' entrance to the cell cycle, increasing the number of NSCs, self-renewal, and synaptic plasticity through the hairy and enhancer of split 1(Hes1)-Notch-1 and NF-κ B/p65 signaling pathways (in activated microglia), and the Notch-PI3K-AKT pathway (in activated astrocytes) [[320], [321], [322], [323]]. Overexpression of Hes1 following the injection of an adenoviral vector carrying Hes1 cDNA impairs adult hippocampal neurogenesis and learning capacity through the suppression of the notch signaling pathway in the DG of the hippocampus after TBI model [324].

Other experiments showed that STAT1 or STAT3 could regulate the maintenance of NSCs in the hippocampus of SCI model which may be correlated with the JNK signaling and NF-κB/p65 pathway [325,326], or astrocyte secretory polypeptides via the JAK-*STAT* pathway [327]. HMGB1, as a non-histone DNA binding protein involved in DNA repair, also regulates both neuroinflammatory cascades in microglia through interaction with HMGB1-TLR4-NF-κ B in microglia and astrocyte, as well as neurogenesis via the HMGB1-RAGE–NF–κ B axis following TBI [328,329]. Inhibition of the TLR4/NF-κB/NLRP3 inflammatory signaling pathway is also associated with neurogenesis and angiogenesis in other neurodegenerative diseases [330]. N-formylpeptide receptor 1 (Fpr1) also promotes survival and neural differentiation in the DG region and decreases acute inflammation through the MAPk- NF-κ B - NLRP3 after TBI [331]. In addition, the compounds involved in the regulation of hippocampal neurogenesis following TBI are specified in Table 3.

**Table 3:** Strategies involved in regulation of hippocampal neurogenesis following TBI.

Molecules	TBI models	Pathways	Properties	Ref.
NPC transplantation	Lateral fluid percussion	Neurotrophin (MNTS1))–Trk	Up regulation of endogenous hippocampal neurogenesis, immune modulatation	[281]
N-acetyl serotonin (NAS)	CCI model	TrkB/PI3K/Akt/(CREB)	Increase of hippocampal neurogenesis, CREB phosphorylation,	[19]
Phosphorylation assessment	CCI model	Akt/GSK- 3beta/beta-catenin	Increase of Neuronal survival and decrease apoptosis	[293]
Electroacupuncture	*CCI model*	inhibition of both TLR4/Myd88/NF-ĸB- & TLR4/TRIF/NF-ĸB	enhancement of double labeled newborn neurons in the SGZ	[34,332]
Curcumin	CCI model	BDNF/Trkb/PI3K/Akt pathway	Enhanced hippocampal DG neurogenesis and decreased hippocampal neuroinflammation	[37]
miR-124enriched exosomes	CCI model	TLR4 pathway	Enhance M2 polarization of Microglia and Hippocampus neurogenesis (NSCs differentiation and proliferation)	[306]
S1PRs	CCI model	MEK/Erk pathway	increase proliferation of NSCs in hippocampus	[39]
VEGF	CCI model	PI3K –Akt, Raf/MEK/ERK pathways	promotes cell survival	[310]
simvastatin	CCI model	PI3K/AKT/GSK-3β/CREB pathway	increase proliferation of NSCs in hippocampus	[333]
NKCC1	CCI model	Raf/MEK/ERK	regulate ion homeostasis, cell proliferation and neuroblast migration	[309]
Survivin	lateral fluid percussion	Wnt/β-catenin pathway	Promotion of neurogenesis	[312]
Hes1	lateral fluid percussion	Notch signaling	Decrease neurogenesis of NPCs	[324]
Fpr1	CCI model	MAPk–NF–κ B-NLRP3 pathway	Promote neurogenesis	[331]

Abbreviations: NPC, neural progenitor cell; MNTS1, human multineurotrophin; CCI model, controlled cortical impact model; PI3K, Phosphoinositide 3-kinase; AKT, Akt kinase; CREB, cAMP response element-binding protein; GSK, GlaxoSmithKline; TLR4, Toll-like receptor 4; TRIF, TIR-domain-containing adapter-inducing interferon-β; S1PRs, sphingosine-1-phosphate receptors; VEGF, Vascular endothelial growth factor; NKCC1, Na–K–Cl cotransporter; Hes1, Hes Family BHLH Transcription Factor 1; Fpr1, Formyl Peptide Receptor 1; NLRP3, NOD-, LRR- and pyrin domain-containing protein 3.

## 7 Discussion

The findings of this study clarify the intricate interplay between microglia-induced neuroinflammation and hippocampal neurogenesis following TBI. Microglia, the resident immune cells of CNS, play a pivotal role in the inflammatory response and can exert both neuroprotective and neurotoxic effect, depending on their phenotypic polarization [334].

One of the key mechanisms highlighted in this study is the involvement of various signaling pathways in regulating microglial activation and neuroinflammation. The JAK/STAT/SOCS pathway, known for its role in anti-inflammatory responses, emerges as a crucial regulator for microglial function [335]. SOCS proteins, such as SOCS1 and SOCS2, act as negative regulators of this pathway, influencing macrophage polarization and function. Modulating the expression or activity of these proteins could potentially shift the balance towards more neuroprotective microglial phenotype [336].

The study also emphasizes the importance of the PI3K/Akt/mTOR signaling pathway in mediating anti-apoptotic and anti-autophagy responses following TBI. Several neuroprotective factors, such as dexmedetomidine, tetrahydrocurcumin, and simvastatin, have been shown to exert their beneficial effects through the activation of this pathway [337]. However, it is crucial to strike a balance, as excessive activation of this pathway may lead to undesirable consequences. Another key pathway implicated in microglial pro-inflammatory responses is the Ras-Raf-ERK MAPK pathway. Modulating the activity of this pathway could potentially attenuate the detrimental effects of neuroinflammation on hippocampal neurogenesis [338].

Interestingly, the study highlights the role of mitochondrial dysfunction in the activation of the NLRP3 inflammasome, a key mediator of the inflammatory response. Mitochondrial dysfunction can lead to the release of mtDNA, ROS, and cardiolipin, which can activate the NLRP3 inflammasome, triggering the release of pro-inflammatory cytokines such as IL-1β and IL-18 [339,340]. Targeting this pathway could represent a promising therapeutic strategy for mitigating neuroinflammation and promoting neuronal repair. Furthermore, the study highlight the complex interplay between microglia and hippocampal neurogenesis. While pathologically activated microglia can inhibit neurogenesis through various mechanisms, such as impaired phagocytosis, dysregulated synaptic maintenance, and secretion of inflammatory factors [42]. The study also suggests that a moderate reducing in microglial activation may not be sufficient to support neurogenesis. This underscores the need for a balanced approach in modulating microglial activity to promote neuronal repair and regeneration.

It is important to note that the M1/M2 paradigm of microglial polarization is an oversimplification, and there exists a spectrum of intermediate phenotypes. Future research should aim to characterize these intermediate states and their specific roles in neuroinflammation and neurogenesis.

## 8 Conclusion

Microglia are associated with hippocampal structural and physiological changes and cognitive impairment after TBI. Depending on their phenotypic polarization (M1 or M2), they can either enhance neuroprotective and neurogenesis processes or inhibit neuronal repair and exacerbate cell damage. Activation of microglia following TBI occurs through several molecular pathways, including mitochondrial dysfunction. Therefore, factors that inhibit the signaling pathways of microglial activation, including the improvement of mitochondrial dysfunction, can be a suitable treatment strategy for hippocampal disorders caused by TBI.

## Data availability

The authors confirm that the data supporting the findings of this study are available within the article.

## CRediT authorship contribution statement

**Seyedeh Parisa Navabi:** Investigation, Data curation. **Firuzeh Badreh:** Writing – original draft, Validation. **Maryam Khombi Shooshtari:** Writing – original draft, Investigation. **Somayeh Hajipour:** Writing – review & editing. **Sadegh Moradi Vastegani:** Writing – review & editing, Writing – original draft, Supervision, Project administration. **Seyed Esmaeil Khoshnam:** Writing – original draft, Visualization, Validation.

## Declaration of competing interest

The authors declare that they have no known competing financial interests or personal relationships that could have appeared to influence the work reported in this paper.
